# Individual characteristics in arts management careers: investigating the highly sensitive person scale on motivation to lead

**DOI:** 10.3389/fpsyg.2024.1392412

**Published:** 2024-07-18

**Authors:** Christian Winther Farstad, Jan Ketil Arnulf

**Affiliations:** ^1^Department of Culture and Communication, Norwegian Business School, Oslo, Norway; ^2^Department of Leadership and Organization, Kristiania University College, Oslo, Norway; ^3^Department of Organization and Leadership, Norwegian Business School, Oslo, Norway

**Keywords:** leadership, personality, field of art, education, development, careers

## Abstract

Research on personality in leadership indicates that self-selection to leadership careers and artistic careers correlates with diverging personality profiles. People in leadership careers traditionally display lower neuroticism and higher conscientiousness than artistic individuals. In between, there are individuals entering arts management careers. To study these individuals directly, we collected Norwegian data from 91 musical theater students and 102 arts management students and compared with 109 business management students. As expected, conscientiousness and neuroticism predicted artistic careers against business management careers, aligned with the “arts for arts’ sake” myth of artists. Interestingly, arts management careers were not different from artistic careers. They weren’t more motivated to take on leadership roles than performing artists either. However, the Highly Sensitive Person Scale indicated that narrower traits of sensitivity predicted higher levels of motivation to lead in many artists. Some arts and arts management students seem to bring unique talents into forms of leadership particularly useful for artistic organizations. Our findings are discussed in terms of how leadership characteristics operate in the field of art, and the effect of domain-specific characteristics in this setting.

## Introduction

Artistic industries have historically observed a sharp division of labor between the roles of artistic performer and the manager in charge of money and operations (Landry, 2011; Järvinen et al., 2015; Byrnes, 2022). While any theater, orchestra, ensemble, band or studio may be viewed in one perspective as a business in need of management, artistic ideals and career developments seem to have followed the motto “art for art’s sake” (Bourdieu, 1993, 1996). Here, art should only be created for the purpose of art itself, and all other motives, such as the desire to earn money, become famous or serve political interests are deemed as suspect. Bourdieu (1993) has even characterized the artistic field as the economic world reversed.

One practical outcome of this tradition is a lack of training opportunities and career pathways for aesthetic practitioners who want to develop their capabilities for management in their industry. Nevertheless, the field of art seems to be confident in attracting and fostering their future leaders as they have a history of recruiting their leaders among themselves. At the same time, individuals entering as leaders in the arts have typically been described as figures with great affinity for the rules of art but who neglect financial and administrative issues (Røyseng, 2008). Therefore, the division of labor could have differential aspects rooted in psychological dispositions. In terms of personality traits, performing artists (i.e., musicians) are found to be characterized by their higher levels of neuroticism and lower levels of conscientiousness (Bandi et al., 2017, 2023; Vaag et al., 2018; Fernholz et al., 2019; Rose et al., 2019; Gjermunds et al., 2020; Kuckelkorn et al., 2021), which is the opposite of what are found in leaderlike individuals (Badura et al., 2022; Galvin et al., 2023; Gardner et al., 2024). This seems somewhat corroborated by urban myths, outing artistic individuals as creative “prima donnas” with erratic tempers and a dislike for conventional, orderly lifestyles, but could as well illustrate how the process of converting individuals from “artist” to “leader” involves obstacles.

Such pictures of artistic individuals may be partly rooted in exaggerated stereotypes, partly in industrial traditions leaving the trivial organizational work to non-performing staff. On closer scrutiny, performing artists could have more interest in and talent for leadership than the stereotypes suggest as they are most often self-disciplined, intrinsically motivated professionals with strong quality requirements for their own productions. Moreover, acts of performance are also strong acts of communication that frequently depend on the concerted efforts of groups. In fact, the orchestra conductor has frequently been invoked as a leadership prototype, warranted or not (Jansson, 2013; Mintzberg, 2016).

The purpose of this study is to explore personality traits involved when individuals enter arts management careers, attempting to identify facets with differential effects on leadership motivation in the field of art. Specifically, we want to look at how personality traits, and motivation to lead, differentiate between artistic and business students. Then, arts management students will be compared with artistic students in terms of how their personality traits provide leadership talent and how their career choice provide motivation to lead. In what follows, we will outline how personality is involved in early choices of career and education. Moreover, we argue that different professional educations are vary in their cultural endorsement of leadership as part of a professional identity. Finally, we explore how individuals crossing the line between arts and management may have talent for leadership hidden under their artistic mantle. Our focus will be on sensory processing sensitivity as a narrower personality trait associated with artistic individuals.

## Theory and hypotheses

The cultural value labeled “art for art’s sake” stems from the profound tradition to separate art and economy in culture economics and sociology (Bourdieu, 1993). Taken as an ideology, this motto views art as autonomous and independent from the economic sphere. Work extraneous to the artistic activity itself, such as business or finance, is even viewed with suspicion (Bourdieu, 1996). To sharpen the division, higher arts education is, to a very limited degree, offering knowledge about leadership and organizing business (Byrnes, 2022). Therefore, artistic individuals are known for their strong artistic identities, including elements where leadership is perceived as threatening the artistic endeavor (Bennett and Bridgstock, 2015; Bridgstock et al., 2015; Bridgstock and Cunningham, 2016). On this basis, individuals who make choices of education in the pursuit of artistic careers might be less talented in, or motivated for, traditional leadership roles. As opposed to artistic careers, educational tracks in business prepare and groom students for managerial roles in charge of organizations. Courses in economy, marketing, strategy, decision making, and leadership are staple ingredients in the syllabi of business schools. Therefore, business individuals who make choices of education in the pursuit of business careers might be more talented in, and motivated for, traditional leadership roles.

As the arts have increasingly emerged as industries of great economic value to society, the need to plan and manage artistic businesses has been picked up by business schools. Arts management programs have become widespread to attract individuals with an aspiration for leadership in the field of art (Laughlin, 2017; Byrnes, 2022). Arts management programs typically offer domain-specific courses emphasizing cultural policy, specific organizational and commercial structures for the different branches in the field of arts (music, fine art, theater, etc.), and topics in law, in addition to traditional business courses such as marketing, economy, strategy and finance. While arts leadership programs provide domain-specific models and concepts to professionalize the management function in the field of art, they often attract individuals with previous background and experience from the artistic field. Some programs even put higher arts education as a prerequisite for admission (Elstad and Jansson, 2020). As a result, individuals in arts management careers often report a strong sense of passion for the art s as a key driver for their career (Richardson et al., 2017).

Whereas stereotypical differences between individuals entering artistic careers and individuals entering business management careers could be categorized in populistic terms such as “natural born artists” or “natural born leaders”, individuals entering arts management careers are somewhat more diffuse. To study such differences, the following chapters will explain how personality traits might explain artistic and leadership talent in arts management individuals, and how motivation to lead might explain how arts management careers stimulate their drive to pursue activities associated with leadership.

### Personality traits in careers

Personality traits are assumed to represent relatively stable dispositions across life (e.g., Damian et al., 2019) and have been shown to influence vocational interests and career choices (Volodina et al., 2015; Vedel, 2016; Stoll et al., 2020). While vocational interests are found in relation to specific career choices (e.g., Volodina and Nagy, 2016), personality traits describe how such interests and career choices fulfill deeper needs in individuals (Ackerman and Beier, 2003). As occupations tend to differ in how they fulfill such needs, personality traits tend to form occupational stereotypes. The interaction between personality traits, vocational choices and careers is described in the attraction-selection-attrition model (Schneider et al., 2012). Here, stereotypes are argued to be a result of occupational tendencies to attract, select, and retain an increasingly homogeneous group of individuals to operate effectively and pursue its identity.

In terms of individuals associated with leadership, personality traits have a long tradition of interest among researchers. As there was less consensus regarding personality traits in the early years, the trait-approach faced skepticism dominated by its lack of consistency. Later, the five-factor model become recognized for categorizing personality traits more consistently around the five traits: neuroticism, extraversion, openness to experience, agreeableness, and conscientiousness . In their meta-analysis, Judge et al. (2002) found quite substantial effects between four of the traits and individuals typically seen as leaderlike. The multiple correlation for leadership emergence was .53, represented in neuroticism (corrected *r* = −0.24), extraversion (corrected *r* = 0.33), openness to experience (corrected *r* = 0.24), and conscientiousness (corrected *r* = 0.33). Characteristics describing leaderlike individuals are likely to predict leadership across a number of criteria and sectors (Badura et al., 2022), also in individuals typically entering business management careers (Lounsbury et al., 2009).

While the fascination for personality traits and leadership has attracted scholars for decades, they seem to be less enthusiastic about studying individuals in the field of art (Byrnes, 2022). As of today, no known studies provide any conclusive overview or meta-analysis of personality traits associated with individuals in artistic careers. Still, there are studies describing single disciplines with artistic individuals as higher in neuroticism and openness to experience and lower in conscientiousness. For example, several types of performing artists such as musicians, dancers and actors have been reported as higher in neuroticism and openness to experience (Nettle, 2006; Bandi et al., 2017, 2023; Butkovic and Dopudj, 2017; Vaag et al., 2018; Fernholz et al., 2019; Gjermunds et al., 2020; Kuckelkorn et al., 2021; Christensen et al., 2024). Furthermore, these individuals have been described as lower in conscientiousness (Fink and Woschnjak, 2011; Bandi et al., 2017; Vaag et al., 2018; Rose et al., 2019; Gjermunds et al., 2020).

Based on the considerations above, we believe that individuals in artistic careers differ from individuals in business management careers in terms of personality traits such as neuroticism and conscientiousness. As being relatively stable psychological dispositions (e.g., Damian et al., 2019), we believe their personality traits come prior to their choice of their respective careers. Therefore, we also assume that personality traits are able to differentiate individuals in terms of their career choices.

*H1a: Individuals choosing careers in performing arts are predicted by higher levels of trait neuroticism, significantly different from business careers.*

*H1b: Individuals choosing careers in performing arts are predicted by lower levels of trait conscientiousness, significantly different from business careers.*

No known studies have previously investigated personality traits in individuals entering arts management careers. Based on studies reporting arts management programs attracting individuals with previous background and experience in the field of art (Elstad and Jansson, 2020), it is reasonable to believe they share characteristics in terms of personality traits as well.

*H1c: Individuals choosing careers in arts management are not significantly different from their counterparts in the performing arts in terms of their personality traits.*

### Motivation to lead in careers

To pursue a career in management is a motivated choice, not an innate effect of traits (Auvinen et al., 2020). The path toward this decision has been mapped with a construct termed Motivation to Lead (MTL; Chan and Drasgow, 2001). This construct envisages leadership motivation as a state, not a trait, and hypothesizes that leadership is developed through engagement in activities associated with leadership. This allows individuals to acquire the social skills and knowledge required for leading and to develop a personal leadership style (Chan and Drasgow, 2001). Careers in leadership are thereby determined by a multitude of contextual factors spanning from professional experiences and backward through education to parenting style, attachment style, education, leisure activities, self-regulation, vocational interests and identity (Chan et al., 2000; Murphy and Johnson, 2011; Avolio et al., 2014; Guillén et al., 2015; Joo et al., 2018; Kragt and Day, 2020). In short, “key to this approach are the assumptions that one’s leadership skills and leadership style are learned and that motivation to lead can be changed” (Chan and Drasgow, 2001, p. 482).

The concept of MTL is built on three underlying components derived from motivational theory, the first being an intrinsic affective-identity component, the second a social normative component and the third a non-calculative component (Chan and Drasgow, 2001). Affective-Identity MTL reflects the fact that some people just like to lead. As an intrinsic motivational facet, it is associated with the pleasure in handling the tasks involved in leadership. The sub-factor also relates with leadership self-efficacy, core self-evaluation, and general self-efficacy. Individuals high on this sub-factor have typically more past leadership experience than their peers as well (Chan and Drasgow, 2001). Social-normative MTL reflects social norms and the fact that some want to lead from a sense of duty and obligation to take responsibility. Non-calculative MTL reflects instrumentality and the fact that some bring in assumptions about the cost of leading relative to the benefits. Individuals high in non-calculative MTL wish to keep social surroundings harmonious, an expression of altruism instead of egoism (Chan and Drasgow, 2001).

The developmental nature of MTL highlights how vocational domains offer or block opportunities for leadership experience, in short how an educational track grows future leaders (Astin, 1993; Komives et al., 2009). As described initially, the prevalent ideology praising “art for art’s sake” (Bourdieu, 1993, 1996) has led to a situation where higher arts education rarely offers knowledge about leadership and organizing business (Byrnes, 2022), thereby preventing leadership motivation from developing. This situation is quite contrary to the context offered to business management students, where the business schools try to outshine each other in the extent to which they claim to develop “tomorrow’s leaders” (Khurana, 2010). Therefore, it is reasonable to believe that individuals in artistic careers differ from individuals in business management careers in terms of their MTL.

*H2a: Performing arts careers are characterized by lower levels of motivation to lead, significantly different from business management careers.*

Also individuals in artistic careers tend to end up working in creative industries and other industries (Bridgstock et al., 2015) where they are exposed to the necessities of management and leadership, learning the trade as an opportunity for future careers in the field. Based on the concept of MTL, such situations are examples of activities potentially increasing individuals’ motivation to pursue such careers. Even individuals with stereotypical artistic characteristics could develop leadership motivation and pursue a career in arts management. Therefore, it is reasonable to believe that individuals in arts management careers differ from individuals in artistic careers in terms of their motivation to lead.

*H2b: Arts management careers are characterized by higher levels of motivation to lead, significantly different from performing arts careers.*

### Artistic characteristics in motivation to lead

As argued above, we expect personality traits to represent dispositions that come prior to career choice, and vocational practice will in turn influence leadership motivation. However, several studies have investigated a relationship between MTL and personality traits, indicating that leaderlike characteristics tend to be involved in individuals typically becoming motivated for leadership careers (Chan and Drasgow, 2001; Badura et al., 2020). Whereas this could indicate another obstacle for artistic individuals entering management careers, scholars have recommended implementing narrower personality constructs to improve how we understand such relationships in specific environments (Paunonen et al., 1999; Bainbridge et al., 2022). On this basis, there might be personality facets at play that drive individuals toward artistic careers, which also can fill in as assets for developing leadership motivation. If we can find a more nuanced dynamism behind artistic careers, it will be possible to develop a more suitable rationale for supporting artistic individuals taking charge in artistic industries.

#### Sensory Processing Sensitivity

As a more general characteristic, sensitivity has regularly been used to describe artistic individuals. For example, studies have found musicians to be more sensitive to small and subtle changes in sound such as frequency, pitch and intervals (Amir et al., 2003; Micheyl et al., 2006; Zarate et al., 2012; Carey et al., 2015), and sensitive and responsive to the good and beautiful (Güsewell and Ruch, 2014). Also, dancers have been found to be more emotional sensitive toward others (Izountouemoi and Esteves, 2023), and sensitive to body movements in others (Orlandi et al., 2017).

As a personality trait, sensory processing sensitivity is defined as involving deeper processing of stimuli across a very wide variety of situations, supported by a greater response to both positive and negative stimuli that motivates learning and thus leads to more successful responses in future similar situations (for a comprehensive review, see Greven et al., 2019). The Highly Sensitive Person Scale (HSPS; Aron and Aron, 1997) was developed to purportedly measure stable individual differences in sensory processing sensitivity. Individuals with high scores on the scale tend to notice more subtle stimuli in their environment and are more easily aroused by this.

The HSPS is often referred to in terms of negative emotions through reports of anxiety, agoraphobia, social anxiety, depression, mental health, avoidant personality disorder, borderline personality disorder, and anger (Ahadi and Basharpoor, 2010; Booth et al., 2015; Greven et al., 2019; Jagiellowicz et al., 2020). In a study, Aron and Aron (1997) found highly sensitive persons prone to reactions such as starting to cry, sensitivity for daylight, sensitivity for alcohol, life in the countryside, intense feelings of love, and need for time alone.

To better understand the psychometric mechanisms in the HSPS, Smolewska et al. (2006) suggested three distinct sub-factors of the scale. The first, ease of excitation (EOE) is described as to what extent individuals are overwhelmed by external and internal demands. This factor also seems to be the one most associated with neuroticism and is predicted by Gray’s (1991) behavioral inhibition system. The second sub-factor, low sensory threshold (LST) reflects how easily individuals are aroused by external stimuli, such as bright light, loud noise, and scarce fabrics. Along with EOE, this sub-factor is associated with neuroticism. The two sub-factors are also found negatively related with extraversion (Smolewska et al., 2006; Sobocko and Zelenski, 2015; Listou Grimen and Diseth, 2016). The last sub-factor, aesthetic sensitivity (AES), in general reflects one’s awareness of aesthetics in the surroundings, as well as depth of processing, which refers to processing stimuli more thoroughly. This sub-factor seems less associated with neuroticism, but is the only one of the three positively related with openness to experience (Smolewska et al., 2006; Sobocko and Zelenski, 2015; Listou Grimen and Diseth, 2016). Aesthetic sensitivity is also predicted by Gray’s (1991) behavioral activation system.

Sensory processing sensitivity is related to performing artists through the path of creativity (Bridges and Schendan, 2019). Individuals with an perchant for creativity and the arts are also found associated with this trait (Aron, 2013). Another study found musicians higher in the HSPS when compared with non-musicians (Lindholm, 2015). They also reported this sensitivity positively related to self-reported musician self-efficiency. It was even argued that sensory processing sensitivity is a part of the concept of musicality and artistry. In contrast with several other outcomes, it seems like sensitivity may benefit artistic individuals in the field of art.

#### Sensory processing sensitivity in leadership

Studies on HSPS have been applied into the context of organizations as well, often in terms of understanding negative outcomes. Criteria such as sense of coherence, lack of self-confidence, alienation, work stress, work displeasure, exhaustion, disengagement, and need for recovery seem associated especially with EOE and LST (Golonka and Gulla, 2021). Another study suggested that EOE and LST cause vulnerability in the work context as they amplify the relation between job demands and emotional exhaustion (Vander Elst et al., 2019). Interestingly, AES seems to be associated with more positive criteria, such as being able to influence, settle, and change things in the environment (Vander Elst et al., 2019), as well as a protective factor against exhaustion and burnout (Golonka and Gulla, 2021).

The HSPS has never been investigated in possible conjunction with MTL. Based on previous studies indicating that MTL emerges in people with personality patterns predictive of leadership emergence, we want to outline how this might work out:

First, we assume individuals scoring high on EOE might easily get overwhelmed in leadership positions. The mere idea of such positions, associated with responsibility, stress, decision-making, and information overload, might erode the motivation to lead in subjects scoring high on EOE.

*H3a: The MTL sub-factors are negatively predicted by EOE*

Conversely, through its relationship with trait openness to experience, we assume AES might provide a resource in leadership motivation. The heightened awareness of details in one’s surroundings, followed by a depth of processing stimuli could result in a better overview, a more interested immersion in situations that might result in better decisions and actions. Individuals high in aesthetic sensitivity might therefore have strong experiences about situations, resulting in more leadership motivation.

*H3b: The MTL sub-factors are positively predicted by AES*

Individuals high on LST tend to get more easily aroused by external stimuli, which might more easily turn into negative outcomes such as stress, exhaustion, and burnout. As leaders more often are described as being in the middle of people and handling high amount of complex information from several sources at the same time, we assume that individuals high on LST might be more skeptical to become associated with concept of leadership, resulting in less leadership motivation.

*H3c: The MTL sub-factors are negatively predicted by LST*

## Materials and methods

### Participants

*Artistic Students*. In total, 91 Norwegian students (age average = 22, SD = 2,2; male = 23 female = 68) from the Musical Theater University College (bachelor level) responded to the survey for this study. The survey was distributed and filled out as a class exercise. All students have previous performance experience from singing, dancing and acting before entering the school. For most of them, the university college is their first step into a professional career as performing artists. These students were not exposed for topics in leadership or management during their education program.

*Arts Management Students*. In total, 102 Norwegian students (age average = 24, SD = 3,6; male = 29, female = 73) from the arts management program (bachelor level) at the Norwegian Business School responded to the survey for this study. The survey was distributed and filled out as a class exercise. Students in this program typically have a background, and experience in, the field of art. The program is designed for individuals who plan to do administrative and managerial work in the field of arts or close industries. As part of their educational program at a business school, these students are exposed for leadership in lectures, literature, guest speakers, excursions, and other social events relevant for business school students.

*Business Students*. In total, 109 Norwegian students (age average = 24,3, SD = 4,9; male = 27, female = 82) from the business management program (bachelor level) at the Norwegian Business School responded to the survey for this study. The survey was distributed and filled out as a class exercise. The business management students are not expected to typically have any common interest in arts but share an interest in a career in business administration. As part of their educational program at a business school, these students are exposed for leadership in lectures, literature, guest speakers, excursions, and other social events relevant for business school students. The total sample of 302 students (male = 79, female = 223) had a mean age of 23,4 (SD 3,9).

### Measures

Each of the scales was explored through a confirmatory factor analysis (CFA). Fit indices for the initial model are presented followed by modified models (RMSEA; CFI; SRMR). Modification of the scales was based on the modification indices reported in the analysis (Lisrel 8.80).

*Personality traits*. Personality was measured using the five-factor scale Mini-IPIP (Donnellan et al., 2006), measuring trait neuroticism, extraversion, intellect, agreeableness, and conscientiousness. The scale was translated properly back-forward for Norwegian use (Keener et al., 2023). The items were measured on a 5-point Likert-scale. A CFA was applied to the set of 20 items, indicating a somewhat acceptable fit; *x*^2^(160) = 390.65 *p* = 0.000; RMSEA = 0.067; CFI = 0.92; SRMR = 0.082. Based on the modification indices, correlations between pairs of items were suggested (C1-C4, A2-A3, I2-I3, N1-N4) resulting in a justified and more acceptable model; *x*^2^(156) = 318.52 *p* = 0.000; RMSEA = 0.059; CFI = 0.94; SRMR = 0.077. The justified model of 20 items loading on its respective five factors was used in the analysis.

*Sensory Processing Sensitivity*. Sensory processing sensitivity was measured using the Highly Sensitive Person Scale (Aron and Aron, 1997) with the three-factor solution suggested by Smolewska et al. (2006). The scale was translated properly back-forward for Norwegian use (Keener et al., 2023). The total scale consists of 27 items measuring sensory processing sensitivity on a 7-point Likert-scale. A CFA was applied to the set of 27 items, indicating a somewhat poorer fit; *x*^2^(321) = 1009.10 *p* = 0.000; RMSEA = 0.084; CFI = 0.91; SRMR = 0.13. Based on the modification indices, five items were deleted from the model due to cross-loadings (EOE6, AES7, AES8, LST5, LST6). It also suggested errors in correlation between one pair of items (EOE2-EOE3) resulting in a justified and more acceptable model; *x*^2^(205) = 397.88 *p* = 0.000; RMSEA = 0.056; CFI = 0.95; SRMR = 0.080. The justified model of 22 items loading on its respective three factors was used in the analysis.

*Motivation to Lead*. Leadership motivation was measured using the Motivation to Lead scale (Chan and Drasgow, 2001), including the three sub-factors, affective-identity, non-calculative, and social-normative. The scale was translated properly back-forward for Norwegian use (Keener et al., 2023). The total scale consists of 27 items measuring leadership motivation on a 7-point Likert-scale. A CFA was applied to the set of 27 items, indicating a somewhat acceptable fit; *x*^2^(321) = 759.49 *p* = 0.000; RMSEA = 0.067; CFI = 0.95; SRMR = 0.10. Based on the modification indices, two items were deleted from the model due to cross-loadings (NC4, NC9) resulting in a justified model; *x*^2^(272) = 600.66 *p* = 0.000; RMSEA = 0.063; CFI = 0.96; SRMR = 0.080. The justified model of 25 items loading on its respective three factors was used in the analysis.

## Results

See Table 1 for correlations and reliability coefficients. All the means were standardized for further analysis. Table 2 reports means, standard deviation and ANOVA for the scales used in the study. The table also compares the three samples: artistic students, arts management students, and business management students. Table 2 reports differences between artistic students and business management students, whereas artistic students differ significantly from business management students in regard to higher neuroticism, higher intellect, higher agreeableness, and lower conscientiousness. Further, Table 2 provides support for hypothesis 1c, assuming that individuals in artistic careers and individuals in arts management careers are not significantly different from each other. As a consequence, Table 2 also reports how arts management students are significantly different from business management students in terms of higher levels of openness to experience and agreeableness and lower levels of conscientiousness.

**TABLE 1:** Inter-scale correlations.

		1	2	3	4	5	6	7	8	9	10	11
1	N	*(0.72)*										
2	E	−0.068	*(0.76)*									
3	I	−0.074	0.202**	*(0.71)*								
4	A	0.155**	0.29**	0.296**	*(0.75)*							
5	C	−0.193**	0.059	0.033	0.081	*(0.69)*						
6	EOE	0.529**	−0.189**	−0.141**	0.108	−0.29**	*(0.78)*					
7	AES	0.104	0.165**	0.547**	0.491**	0.088	0.072	*(0.69)*				
8	LST	0.474**	0	0.025	0.208**	−0.076	0.571**	0.201**	*(0.72)*			
9	AI	−0.024	0.452**	0.21**	0.129*	0.255**	−0.298**	0.141*	0.017	*(0.91)*		
10	NC	−0.042	0.087	0.052	0.253**	0.112	−0.164**	0.116*	−0.055	0.108	*(0.81)*	
11	SN	−0.103	0.332**	0.096	0.051	0.182**	−0.179**	0.125*	−0.067	0.507**	−0.02	*(0.68)*

**p < 0.01.

*p < 0.05.

N, neuroticism; E, extroversion; I, intellect; A, agreeableness; C, conscientiousness; EOE, ease of excitement; AES, aesthetic sensitivity; LST, low sensitivity threshold; AI, affective-identity MTL; NC, non-calculative MTL; SN, social-normative MTL.

**TABLE 2:** Descriptive for all variables in the three samples.

	Artistic		Arts Management		Business Management		
	Mean	SD	Mean	SD	Mean	SD	F(2, 299)
N	3.15^c^	0.79	2.92	0.85	2.69^a^	0.83	7.42
E	3.37	0.93	3.29	0.82	3.49	0.83	1.35
I	3.92^c^	0.84	3.98^c^	0.74	3.62^ab^	0.81	6.05
A	4.47^c^	0.57	4.42^c^	0.63	4.1^ab^	0.65	11.09
C	3.24^c^	0.89	3.47^c^	0.78	3.75^ab^	0.70	10.67
EOE	4.49^c^	0.82	4.41^c^	0.89	3.85^ab^	1.03	14.65
AES	5.78^c^	0.66	5.87^c^	0.77	5.22^ab^	0.91	20.75
LST	3.64^c^	1.22	3.41	1.22	3.15^a^	1.33	3.66
AI	4.25^c^	1.4	4.47	1.27	4.76^a^	1.08	4.15
NC	4.97	1.3	4.78	1.05	5.01	1.04	1.26
SN	4.15	0.98	4.05^c^	0.86	4.39^b^	0.73	4.21*

Total students, *n* = 302; Artistic (careers), *n* = 91; Arts Management (careers), *n* = 102; Business Management (careers), *n* = 109. N, neuroticism; E, extroversion; I, intellect; A, agreeableness; C, conscientiousness; EOE, ease of excitation; AES, aesthetic sensitivity; LST, low sensory threshold; AI, affective-identity; NC, non-calculative; SN, social-normative. Tukey:

^a^ different from artistic students;

^b^ different from arts leadership students;

^c^ different from business students.

Further, hypothesis 2a assumed that individuals in artistic careers differ from individuals in business management careers in terms of lower levels of MTL. The results in Table 2 render only partial support for this hypothesis: the two groups only differ significantly in affective-identity MTL where the artistic students score lower. Hypothesis 2b assumed that individuals in arts management careers differed from individuals in artistic careers in terms of higher levels of MTL. Table 2 indicates no support for this hypothesis, as the two groups do not differ in any of the three sub-factors of MTL. With the lowest mean between the samples, arts management students differ from business management students on social-normative MTL.

Whereas Table 2 reports differences between the three groups, hypothesis 1a and 1b assumed personality traits to predict the individuals in artistic careers. To test these hypotheses, a logistic regression analysis (SPSS ver. 26) was conducted. For each of the analyses the target career was plotted as the dependent outcome. Age and gender (male = 1, female = 2) were controlled for in the analysis.

As reported in Table 3, when put against business management careers the choice of artistic careers is predicted positively by neuroticism and agreeableness and negatively by conscientiousness, which gives support for hypothesis 1a and 1b.

**TABLE 3:** Logistic regression: predicting performing arts careers (1) vs business management careers (0).

	β	*SE* β	Wald’s X^2^	*df*	*p*	e^β^ (odds ratio)
Age	−0.262	0.069	14.324	1	0.000	0.769
Gender (female)	−0.261	0.458	0.326	1	0.568	0.770
N	0.428	0.198	4.663	1	0.031	1.534
E	−0.316	0.191	2.742	1	0.098	0.729
I	0.262	0.182	2.082	1	0.149	1.299
A	0.789	0.216	13.317	1	0.000	2.202
C	−0.705	0.189	13.861	1	0.000	0.494
Constant	6.374	1.845	11.936	1	0.000	586.238

(Chi-Square: 72.333(7), *p* = 0.000; 2LL: 203.303; Cox & Snell R Square: 0.303; Nagelkerke R Square: 0.406). N, neuroticism; E, extroversion; I, intellect; A, agreeableness; C, conscientiousness.

Whereas Table 2 provided support for hypothesis 1c, we conducted a similar logistic regression analysis with the aim of predicting arts management careers as opposed to artistic careers. As reported in Table 4, none of the personality traits were able to predict arts management careers as opposed to artistic careers. This somewhat strengthens the support for hypothesis 1c.

**TABLE 4:** Logistic regression: predicting arts management careers (1) vs performing arts careers (0).

	β	*SE* β	Wald’s X^2^	*df*	*p*	e^β^ (odds ratio)
Age	0.223	0.063	13.114	1	0.000	1.250
Gender (female)	0.111	0.395	0.080	1	0.778	1.118
N	−0.248	0.167	2.205	1	0.138	0.780
E	−0.134	0.166	0.647	1	0.421	0.875
I	0.012	0.173	0.005	1	0.945	1.012
A	−0.093	0.192	0.236	1	0.627	0.911
C	0.285	0.154	3.429	1	0.064	1.330
Constant	−5.048	1.553	10.567	1	0.001	0.006

(Chi-Square: 23.371(7), *p* = 0.001; 2LL: 243.557; Cox & Snell R Square: 0.114; Nagelkerke R Square: 0.152). N, neuroticism; E, extroversion; I, intellect; A, agreeableness; C, conscientiousness.

For similar reasons, we conducted a logistic regression analysis with the aim of predicting arts management careers against business management careers. As reported in Table 5, lower levels in extraversion and conscientiousness and higher levels in openness to experience and agreeableness predicted the arts management careers.

**TABLE 5:** Logistic regression: predicting arts management careers (1) vs business management careers (0).

	β	*SE* β	Wald’s X^2^	*df*	*p*	e^β^ (odds ratio)
Age	−0.063	0.043	2.121	1	0.145	0.939
Gender (female)	−0.386	0.388	0.988	1	0.320	0.680
N	0.288	0.173	2.770	1	0.096	1.334
E	−0.518	0.179	8.345	1	0.004	0.595
I	0.565	0.186	9.215	1	0.002	1.760
A	0.608	0.183	11.042	1	0.000	1.836
C	−0.616	0.183	11.405	1	0.000	0.540
Constant	2.311	1.264	3.344	1	0.067	10.086

(Chi-Square: 55.751(10), *p* = 0.000; 2LL: 236.525; Cox & Snell R Square: 0.232; Nagelkerke R Square: 0.310). N, neuroticism; E, extroversion; I, intellect; A, agreeableness; C, conscientiousness; AI, affective-identity MTL; NC, non-calculative MTL; SN, social-normative MTL.

To test the hypothesis 3a–3c, a multiple regression analysis (SPSS ver. 26) was conducted to predict each of the three sub-factors of MTL. Age and gender (male = 1, female = 2) were controlled for in the analysis. Table 6 reports all the coefficients in the analysis for each of the three sub-factors. Each sub-factor was analyzed through three models; model 1 reports coefficients for sensory processing sensitivity only; model 2 reports coefficients for the personality traits only; model 3 reports coefficients for both sensory processing sensitivity and the personality traits together.

**TABLE 6:** Multiple regression analysis: predicting three sub-factors of motivation to lead.

	Affective-Identity MTL			Non-Calculative MTL			Social-Normative MTL		
	Model 1	Model 2	Model 3	Model 1	Model 2	Model 3	Model 1	Model 2	Model 3
Intercept	−0.346	−0.073	0.125	−1.194*	−1.145*	−1.189*	0.113	0.445	0.481
Age	0.021	0.022	0.001	0.027	0.064	0.054	−0.001	−0.008	−0.018
Gender	0.056	−0.014	−0.033	0.261**	0.193**	0.219**	−0.028	−0.101	−0.095
EOE	−0.445**		−0.319**	−0.182*		−0.193*	−0.2*		−0.069
AES	0.126*		0.008	0.141*		0.044	0.133*		0.126
LST	0.227**		0.156*	−0.066		−0.069	0.03		0.006
N		0.078	0.155*		−0.123*	−0.016		−0.01	0.005
E		0.434**	0.389**		−0.026	−0.061		0.347**	0.339**
I		0.134*	0.091		0.004	−0.028		0.016	−0.046
A		−0.064	−0.045		0.236**	0.249**		−0.045	−0.082

*N* = 302. EOE, ease of excitation; AES, aesthetic sensitivity; LST, low sensory threshold; N, neuroticism; E, extroversion; I, intellect; A, agreeableness; C, conscientiousness; AI, affective-identity MTL; NC, non-calculative MTL; SN, social-normative MTL.

Hypothesis 3a suggested that the MTL sub-factors were negatively predicted by EOE. As reported in Table 6, both affective-identity MTL, non-calculative MTL, and social-normative MTL are negatively predicted by EOE, which gives full support for the hypothesis. Hypothesis 3b suggested that the MTL sub-factors were positively predicted by AES. As reported in Table 6, all the sub-factors are positively predicted by AES, which gives full support for the hypothesis. Hypothesis 3c suggested that the MTL sub-factors were negatively predicted by LST. As reported in Table 6, the affective-identity MTL is positively predicted by LST, whereas non-calculative MTL and social-normative MTL are not significant. The hypothesis is not supported.

A narrower investigation of the affective-identity MTL in Table 6 indicates that neuroticism changes from weak and insignificant in model 2 to positive and significant in model 3. The inclusion of sensory processing sensitivity might take out variance of neuroticism, and what is left becomes more relevant for predicting leadership. This will be further investigated in the following suppressor analysis.

Based on Horst (1941), a suppressor effect occurs when a variable that is uncorrelated with the criterion nonetheless improves prediction when added to the equation. This pattern occurs when the new variable is positively correlated with a predictor already in the equation. The effect defined as the suppressor variable removes (or suppresses) criterion-irrelevant variance from the initial predictor (Paulhus et al., 2004). As a result, the initial predictor becomes a more efficient predictor, and the suppressor variable represents new explained variance in the equation. Among other models of suppressor effect, Horst’s model is labeled classical suppression (Paulhus et al., 2004).

As reported in Table 1, neuroticism is uncorrelated with affective-identity MTL and is strongly and positively correlated with EOE. As found in Table 6, EOE predicts affective-identity MTL, whereas neuroticism does not. A regression analyses was applied to test neuroticism as a suppressor on EOE in the three student samples.

As reported in Table 7, neuroticism only predicts the affective-identity MTL positively and significantly when EOE is in the equation. The results also report that neuroticism improves the effect of EOE when present in the equation. This suppressor effect seems to be different between the three samples. While the presence of neuroticism improves the effect of EOE in the total sample, the effect of neuroticism on the affective-identity MTL only becomes significant for the individuals in arts management careers. Neuroticism also has an elevated coefficient (not significant) for the individuals in artistic careers, which could indicate this to be an artistic phenomenon.

**TABLE 7:** Regression of affective-identity MTL on neuroticism and ease of excitation in three samples.

			Neuroticism			Ease of Excitation		
	N	r_12_	β	β with EOE	Δ *P*^2^	β	β with N	Δ*R*^2^
Total Sample	302	0.53	−0.02	0.23**	0.11	−0.3**	−0.5**	0.02
Artistic	91	0.34	0.05	0.22	0.09	−0.26*	−0.51**	0.01
Arts Mgmnt	109	0.55	0.08	0.5**	0.23	−0.35**	−0.79**	0.11
Business Mgmnt	102	0.58	−0.1	0.03	0.02	−0.21*	−0.22	−0.01

**p < 0.01.

*p < 0.05.

## Discussion

The purpose of this study was to explore personality traits involved when individuals enter arts management careers, attempting to identify facets with differential effects on leadership potential in the field of art. This is of interest because traits associated with artistic careers have so far been regarded as less compatible with leadership careers. Whereas we do not assume leadership to be “universal”, we investigated arts leadership careers as we believe they are important for understanding the potential for effective leadership in the field of art. The importance of domain-specific abilities is also focused in the initial model of motivation to lead (Chan and Drasgow, 2001). Therefore, we explored in which way nuances in personality traits can identify individuals who are predisposed for artistic careers, while at the same time displaying talents for leadership careers. Hypotheses and results are reported in Table 8.

**TABLE 8:** Summary of hypotheses.

Hypotheses	Results
**H1a**: Individuals choosing careers in performing arts are predicted by higher levels of trait neuroticism, significantly different from business careers.	S
**H1b**: Individuals choosing careers in performing arts are predicted by lower levels of trait conscientiousness, significantly different from business careers.	S
**H1c**: Individuals choosing careers in arts management are not significantly different from their counterparts in the performing arts in terms of their personality traits	S
**H2a**: Performing arts careers are characterized by lower levels of motivation to lead, significantly different from business management careers.	NS
**H2b**: Arts management careers are characterized by higher levels of motivation to lead, significantly different from performing arts careers.	NS
**H3a**: The MTL sub-factors are negatively predicted by EOE	S
**H3b**: The MTL sub-factors are positively predicted by AES	S
**H3c**: The MTL sub-factors are negatively predicted by LST	NS

S, support; NS, not support.

For this purpose, we compared three groups – artistic students, arts management students, and business management students. Underlying this design was the assumption that there is a tendency for self-selection based on personality traits where business management students would score higher on traditional leaderlike traits and artistic students would score higher on traditional artistic traits. In between here, the arts management students are somewhat more diffuse. Our hypotheses envisaged a situation where personality traits would explain how the choice of artistic careers differs from business management careers in terms of higher levels of neuroticism and lower levels of conscientiousness. These hypotheses were confirmed. Other studies reporting similar patterns in relation to creative work support our findings (Clark and DeYoung, 2014). However, personality traits are argued to have relatively stable qualities (Damian et al., 2019) and occur prior to their choice of careers. Therefore, we assumed these characteristics to be a self-selection criterion for entering artistic careers and not a consequence of being in the field of art.

Whereas our assumptions regarding artistic individuals and leaderlike characteristics were supported, it also turned out that the personality configuration of individuals entering arts management careers was not significantly different from the artistic careers. In contrast, arts management students differed from business management students in terms of lower higher levels in openness to experience and agreeableness, and lower levels of conscientiousness and extraversion. As described as being among the strongest traits associated with leadership (Judge et al., 2002), we found the level of extraversion in the arts management students noteworthy. As a trait related with social competence, extraverts tend to be active, energetic, lively, assertive, and less silent and withdrawn (Gough, 1988), which tends to come out as leaderlike behavior perceived by others (Badura et al., 2022). Extraversion was not able to predict artistic careers and is in this study unique in predicting arts management careers. As seen in Table 2, the three student groups were not significantly different in that trait either. Still, arts management students reported the lowest score, indicating that artistic students and business management students might be more similar in this trait than for arts management students. In correspondence with previous research, extraversion is also found as a talent for individuals performing on the stage and expressing themselves in front of others (Nettle, 2006). As a potential talent for both artistic careers and business management careers, individuals in arts management careers could fall between the two. Our study was not able to report a result on this matter, but it could be assumed that individuals lacking the prescribed level of extraversion in the field of art look for other opportunities, such as arts management. This impacts another urban myth, outing arts leaders as unsuccessful artists. At the same time, this could question how extraversion is seen as an characteristic for emerging leaders in settings such as the field of art.

However, we expected MTL to be elevated in the arts management students as a result of their choice of entering a career in leadership and as an effect of being exposed to the leadership literature and professional leadership roles in their education and practice. Higher levels of MTL in individuals in arts management careers could function as well to compensate for their potential lack of leadership talent. Unexpectedly, none of the MTL sub-factors differentiated between the artistic students and the arts management students. One possible reason for this could be the culturally endorsed skepticism toward leadership in the field of art, embracing the ideology of “arts for art’s sake” and leading respondents to distance themselves from self-professing leadership statements. Nonetheless, we expected that their choice to step in and participate in a formal educational program to become arts leaders would increase their MTL. Also, Table 2 provides relevant descriptive results to shed light on the three groups of students. Supporting hypothesis 2b, arts management students were not different from artistic students at any of the MTL sub-factors. At the same time, affective-identity MTL did not differ between arts management students and business management students either. As being associated with both self-efficacy and past leadership experience, affective-identity MTL is a predictor of leaderlike perceptions (Badura et al., 2020). The story seems somewhat different for the other sub-factors, where arts management students score the lowest. On the social-normative MTL, they are even significantly different from the business management students. Again, our study does not provide results to explain these scores, but as described in more dynamic terms (Chan and Drasgow, 2001), they might be more susceptible for contextual influence, such as artistic values. Consequently, their scores might be a result of seeing leadership in the arts more as a “dirty job” less valued in their surroundings.

To explore how more narrow personality traits associated with artistic careers also provides talent for leadership, we made a more detailed exploration of the relationship between sensory processing sensitivity and MTL. Based on the literature, we assumed aspects associated with emotional reactivity to be a hindrance in leadership careers, whereas other aspects, such as aesthetic senses and environmental awareness, were assumed to contribute in leadership careers.

As reported in Table 2, the three sub-factors of HSPS seem to be associated with artistic properties. Thus, the EOE sub-factor explains how artistic individuals are not necessarily talented with respect to leadership careers as they more easily tend to become overwhelmed by their impulses. As such conditions often end in withdrawal from social settings, this limits the individual’s development of social skills to communicate and socialize with others. In comparison, EOE are found related to social and communication deficits (Liss et al., 2005). Such individuals could think of leadership as something exhausting, which could even be something they protect themselves against, resulting in lower MTL.

However, when they enter such careers, the sub-factors AES and LST explain how artistic individuals might bring with them “new” aesthetic resources that could as well be relevant and favorable for development as leaders. First, as this sub-factor denotes the capability to notice subtleties in the environment, individuals high in AES seem to benefit from their increased levels of sensitivity and insight. In addition to having greater attention to detail, such individuals are associated with better communication skills (Liss et al., 2005). These are core qualifications in leadership. Therefore, AES could provide personality resources that increase leader identity and in general more positive honorable attitudes toward leadership. Second, the sub-factor LST are described as more similar to EOE, correlating with depression and anxiety. Therefore, it was not expected that heightened perceptual sensitivity and awareness for subtle stimuli were positively related to MTL. Based on previous findings, we expected such tendencies to be disturbing for leaders in typical complex contexts rich in stimuli. How LST relates to actual leadership behavior is not a part of this study, but it could be relevant to discuss how LST affect individuals in the educational settings. Here, students explore themselves and how they best can benefit from their own resources, such as awareness for subtle stimuli. As situational awareness could be included in descriptions of effective leadership, LST could be perceived as a strength. This argument find support in studies suggesting LST to be a personal resource in organizational situations with more job resources (Vander Elst et al., 2019). It could be questioned how LST will be associated with actual leadership where individuals might have less resources available in their job.

Finally, we found an unexpected suppressor effect between neuroticism and EOE. As described in the results, this could illustrate how narrower facets of personality are able to explain nuances in the broader categories of traits. As a trait negatively related with both leadership emergence and leadership effectivity (Judge et al., 2002), the suppressor situation demonstrates how neuroticism might also hold leadership talent when EOE is controlled for. This also demonstrates how stereotypical pictures of “natural born leaders” and “natural born artists” might be very simplified descriptions of individuals.

## Conclusion

In this study we found no significant differences in personality traits between artistic individuals and arts management individuals. This support the idea that individuals entering arts management careers are more similar to the individuals they are set to manage than to other business managers. This also illustrate how arts management programs attract individuals with different needs in terms of leadership development. In our study, individuals in arts management programs were not more motivated to lead than individuals in performing arts education. This needs to be investigated more in depth, but could capture a structural problem in leadership related to social construction. Further, narrower traits in performing artists did explain how such individuals might feel more easily overwhelmed by external stimuli, but also how they hold personal resources that could help them in developing leadership capabilities to succeed in such careers.

### Practical implications

By comparing individuals in artistic careers and arts management careers we provide an idea of how they differ, which could be useful in terms of identifying and developing future leaders in the field of art. For arts management programs, this information could be useful in describing their candidates and how they differ from both artistic and business students. In terms of MTL, our results indicate a potential problem in such programs, as arts management students seem to fall behind. To fill this gap, courses could be developed to increase MTL specifically in arts management students, as this has proven to affect both leadership identity and MTL (Kragt and Day, 2020). In conclusion, our findings also suggest how individuals entering leadership careers in the field of art have unique talents. What could be different from other concepts of leadership is the potentially negative association with leadership. As a strong determinant for several leadership outcomes, these individuals as well as their behavior might be different, and probably also should be. Narrower aspects could be useful both to be able to find such leaders, but also to understand what types of leaders and types of leadership are being executed in the field of art.

### Limitation

One of the main limitations of this study is the cross-sectional design. The same respondents were put to answer questionnaires at one point in time. At the same time, we argued that personality traits were relatively stable and could be less susceptible to situational influence. Also, variables involving group membership should not be influenced by such bias. For this study, the artistic sample was collected among students at a school for musical theater. Even though this group contains individuals doing dance, acting, and singing, they might differ from other groups in the field of art. Therefore, characteristics might not be representative across the sector.

### Future research

Whereas this study includes individual differences in the field of arts management, there are several questions that should be investigated in this regard. We believe arts management programs should be further investigated in terms of how they are able to promote leadership identities in their students. One relevant question to ask is to what degree such programs bring forward artistic values, such as art for the art’s sake, or provide supplementary values relevant for future leaders. As several personality traits were found related to arts management students, there seem to be some unique patterns in terms of leadership. In particular, the negative association with extraversion is interesting and should be further investigated, also in terms of effective leadership.

Among the first studies to investigate sensory processing sensitivity in leadership, our result might open up for more studies in a phenomenon that has been debated for decades. For example, there are phrases such as “art of leadership” and “leadership as art” describing execution of leadership as something more than simple tasks and activities (Ladkin and Taylor, 2010). Here, leadership is addressed as something irrational rather than rational, and should be described in activities such as feeling, judgment, and sense (e.g., Hansen et al., 2007). This opens for the importance of being able to maintain longer contact with the senses in order to make the most out of the data available (Springborg, 2010).

## Data availability statement

The raw data supporting the conclusions of this article will be made available by the authors, without undue reservation.

## Ethics statement

Written informed consent was obtained from the individual(s) for the publication of any potentially identifiable images or data included in this article.

## Author contributions

CF: Conceptualization, Formal analysis, Investigation, Methodology, Software, Writing – original draft, Writing – review and editing. JA: Conceptualization, Supervision, Writing – review and editing.
